# Global Burden of Multiple Myeloma: A Systematic Analysis for the Global Burden of Disease Study 2016

**DOI:** 10.1001/jamaoncol.2018.2128

**Published:** 2018-05-16

**Authors:** Andrew J. Cowan, Christine Allen, Aleksandra Barac, Huda Basaleem, Isabela Bensenor, Maria Paula Curado, Kyle Foreman, Rahul Gupta, James Harvey, H. Dean Hosgood, Mihajlo Jakovljevic, Yousef Khader, Shai Linn, Deepesh Lad, Lorenzo Mantovani, Vuong Minh Nong, Ali Mokdad, Mohsen Naghavi, Maarten Postma, Gholamreza Roshandel, Katya Shackelford, Mekonnen Sisay, Cuong Tat Nguyen, Tung Thanh Tran, Bach Tran Xuan, Kingsley Nnanna Ukwaja, Stein Emil Vollset, Elisabete Weiderpass, Edward N. Libby, Christina Fitzmaurice

**Affiliations:** 1 Division of Medical Oncology, University of Washington, Seattle; 2 Institute for Health Metrics and Evaluation, University of Washington, Seattle; 3 University of Belgrade, Belgrade, Serbia; 4 Aden University, Aden, Yemen; 5 University of São Paolo, São Paolo, Brazil; 6 Accamargo Cancer Center, São Paolo, Brazil; 7 International Prevention Research Institute, Ecully, France; 8 West Virginia Bureau for Public Health, Charleston; 9 Albert Einstein College of Medicine, Bronx, New York; 10 University of Kragujevac, Kragujevac, Serbia; 11 Center for Health Trends and Forecasts, University of Washington, Seattle; 12 Department of Community Medicine, Public Health and Family Medicine, Jordan University of Science and Technology, Irbid, Jordan; 13 University of Haifa, Haifa, Israel; 14 Postgraduate Institute of Medical Education and Research, Candigarh, India; 15 University of Milano-Bicocca, Milan, Italy; 16 Institute for Global Health Innovations, Duy Tan University, Danang, Vietnam; 17 University Medical Center, Gronigen, the Netherlands; 18 Golestan Research Center of Gastroenterology and Hepatology, Golestan University of Medical Sciences, Gorgan, Iran; 19 Digestive Diseases Research Institute, Tehran University of Medical Sciences, Tehran, Iran; 20 Haramaya University, Haramaya, Ethiopia; 21 Johns Hopkins University, Baltimore, Maryland; 22 Hanoi Medical University, Hanoi, Vietnam; 23 Department of Internal Medicine, Federal Teaching Hospital, Abakaliki, Nigeria; 24 Department of Medical Epidemiology and Biostatistics, Karolinska Institutet, Solna, Sweden; 25 Department of Research, Cancer Registry of Norway, Institute of Population-Based Cancer Research, Oslo; 26 Division of Hematology, University of Washington, Seattle

## Abstract

**Introduction:**

Multiple myeloma (MM) is a plasma cell neoplasm with substantial morbidity and mortality. A comprehensive description of the global burden of MM is needed to help direct health policy, resource allocation, research, and patient care.

**Objective:**

To describe the burden of MM and the availability of effective therapies for 21 world regions and 195 countries and territories from 1990 to 2016.

**Design and Setting:**

We report incidence, mortality, and disability-adjusted life-year (DALY) estimates from the Global Burden of Disease 2016 study. Data sources include vital registration system, cancer registry, drug availability, and survey data for stem cell transplant rates. We analyzed the contribution of aging, population growth, and changes in incidence rates to the overall change in incident cases from 1990 to 2016 globally, by sociodemographic index (SDI) and by region. We collected data on approval of lenalidomide and bortezomib worldwide.

**Main Outcomes and Measures:**

Multiple myeloma mortality; incidence; years lived with disabilities; years of life lost; and DALYs by age, sex, country, and year.

**Results:**

Worldwide in 2016 there were 138 509 (95% uncertainty interval [UI], 121 000-155 480) incident cases of MM with an age-standardized incidence rate (ASIR) of 2.1 per 100 000 persons (95% UI, 1.8-2.3). Incident cases from 1990 to 2016 increased by 126% globally and by 106% to 192% for all SDI quintiles. The 3 world regions with the highest ASIR of MM were Australasia, North America, and Western Europe. Multiple myeloma caused 2.1 million (95% UI, 1.9-2.3 million) DALYs globally in 2016. Stem cell transplantation is routinely available in higher-income countries but is lacking in sub-Saharan Africa and parts of the Middle East. In 2016, lenalidomide and bortezomib had been approved in 73 and 103 countries, respectively.

**Conclusions and Relevance:**

Incidence of MM is highly variable among countries but has increased uniformly since 1990, with the largest increase in middle and low-middle SDI countries. Access to effective care is very limited in many countries of low socioeconomic development, particularly in sub-Saharan Africa. Global health policy priorities for MM are to improve diagnostic and treatment capacity in low and middle income countries and to ensure affordability of effective medications for every patient. Research priorities are to elucidate underlying etiological factors explaining the heterogeneity in myeloma incidence.

## Key Points

**Question:**

What is the burden of multiple myeloma globally and by country, how has it changed over time, and how widely available are treatments for this disease?

**Findings:**

Myeloma incident cases and deaths increased from 1990 to 2016, with middle-income countries contributing the most to this increase. Treatment availability is very limited in countries with low socioeconomic development.

**Meaning:**

Marked variation in myeloma incidence and mortality across countries highlights the need to improve access to diagnosis and effective therapy and to expand research on etiological determinants of myeloma.

## Teaser

This systematic analysis reports incidence, mortality, and disability-adjusted life-year (DALY) estimates from the Global Burden of Disease 2016 study to characterize the burden of multiple myeloma and the availability of effective therapies for 21 world regions and 195 countries and territories from 1990 to 2016.

## Introduction

Multiple myeloma (MM) is a clonal plasma cell neoplasm with substantial morbidity and mortality, characterized by end organ damage—renal impairment, hypercalcemia, lytic bony lesions, and anemia. With the development of better therapies, myeloma has changed from an untreatable ailment to one that is still not curable but treatable with mostly outpatient therapy. Myeloma treatment has improved substantially over the past decade. The first major advance was the development of autologous stem cell transplant (ASCT) in the 1980s and 1990s.^1^ So-called novel agents were first developed in the late 1990s and early 2000s—beginning with the “IMIDs” (thalidomide and lenalidomide) and followed by the proteasome inhibitors (PIs).^2^ More recently, monoclonal antibodies, such as daratumumab and elotuzumab, and histone deacetylating agents, such as panobinostat, have also been approved by the US Food and Drug Administration (FDA).^2^

Myeloma is unique as a cancer because basic diagnostic testing includes only a complete blood cell count with differential, basic metabolic panel; serum calcium, serum and urine protein electrophoresis; and osseous survey, all of which should be accessible in low- and middle-income countries (LMICs).^3,4^ Many advanced tests, such as the serum-free light-chain assay, fluorodeoxyglucose–positron emission tomography (FDG PET) scan, cytogenetics, and fluorescence in situ hybridization (FISH) may not be available. Because of this, resource-stratified guidelines for the diagnosis and treatment of MM have been developed.^4^ Another unique feature of myeloma is that treatment can be delivered almost exclusively in the outpatient setting (with the exception of ASCT).

Standard treatment for myeloma is largely dependent on patient fitness and underlying health status. For those in good health and younger than 70 to 75 years, the preferred treatment for newly diagnosed patients with myeloma comprises a triplet novel agent regimen, typically including an IMID and PI in combination with glucocorticoids, followed by ASCT and maintenance therapy with low-dose IMID or PI.^5,6^ For those unable to undergo ASCT, standard therapy includes induction with novel agents and low-dose maintenance therapy.^7^

Despite improvements in the care of patients with myeloma, these advances have largely delivered better outcomes to patients in high-income countries. In many LMICs, delivery of cancer care is often hindered by lack of access to general and specialized health care, diagnostics, and advanced treatments, like novel agents, radiation oncology, and stem cell transplantation, leading to poor outcomes. In studies of patients with myeloma treated in Nigeria, lack of access to affordable health care, late presentation, and inadequate treatment were suggested as common factors contributing to poor outcomes, with a 5-year survival of only 7.6%.^8,9^

Necessary data to inform health policies with respect to myeloma on a global level, including cancer control and implementation plans, are not widely available. These include data on incidence, mortality, and availability of effective therapies such as IMIDs, PIs, and ASCT. This study therefore aims to describe the global burden of myeloma from 1990 to 2016 by age, sex, and sociodemographic index (SDI) (a summary indicator of income per capita, educational attainment, and fertility) and to describe the availability and/or approval of effective therapies, such as PIs, IMIDs, and ASCT, worldwide. Understanding these factors for myeloma is critical to establish the need for diagnosis and treatment and to demonstrate differences in myeloma incidence, which can foster further research on the underlying etiologies for myeloma.

## Methods

Flowcharts describing the process to estimate mortality, incidence, prevalence, years lived with disability (YLDs), years of life lost (YLLs), and disability-adjusted life-years (DALYs) can be found in eFigures 1 and 2 in the Supplement. Methods for the Global Burden of Disease (GBD) study and the cancer estimation have been described in detail previously.^3,10,11,12^ To estimate myeloma mortality, we used vital registration (VR) system as well as cancer registry (CR) data. Data sources used for myeloma mortality can be found in the GBD source tool (http://ghdx.healthdata.org/gbd-2016/data-input-sources). The VR and CR data were processed in multiple steps. Major adjustments included the redistribution of undefined codes (“garbage codes”) or codes that cannot be considered to refer to underlying causes of death, and mapping of different coding systems to GBD causes.^13^ Codes from the *International Statistical Classification of Diseases and Related Health Problems, Tenth Revision (ICD-10)* mapped to the GBD cause “multiple myeloma” were C88 (“malignant immunoproliferative diseases and certain other B-cell lymphomas”) and C90 (“multiple myeloma and malignant plasma cell neoplasms”). Cancer registry incidence data were transformed to mortality estimates by using separately modeled mortality-to-incidence ratios (MIRs). The VR data were combined with the mortality estimates that were derived from CR MM incidence data and used as input for an ensemble model of mixed-effects linear models and spatiotemporal Gaussian process regression models for MM cause fractions and death rates.^14^ Covariates used in the models can be found in the Appendix (eTable 1 in the Supplement). Myeloma mortality was adjusted to fit into the separately estimated all-cause mortality in a process called “CodCorrect,” which is an algorithm that scales single causes of death to all-cause mortality and child causes to parent causes.^10^ The YLLs were estimated by calculating the difference between a standard life expectancy and the age at death.^13^ Final MM mortality estimates were divided by MIR, resulting in incidence estimates.

Myeloma survival was estimated by scaling GBD locations between a theoretical best-case and worst-case scenario using the MIR as a scaling factor. For each incidence cohort, 10-year prevalence was estimated using the modeled survival. Ten-year myeloma prevalence was divided into 4 sequelae: (1) diagnosis and treatment (7 months),^15^ (2) remission (remaining duration after taking into account the other sequelae), (3) metastatic and/or disseminated phase (37 months),^16^ and (4) terminal phase (1 month). Each sequela prevalence was multiplied with a distinct disability weight, which can be found in eTable 2 in the Supplement, and these were summed to generate YLDs.^13^ DALYs were calculated as the sum of YLDs and YLLs.^11^ Rates are reported per 100 000 person-years with a 95% uncertainty interval (UI) reported in parentheses. For the age standardization, the GBD world population standard was used.^17^ For measuring availability of effective therapies, lenalidomide global approval information as of 2016 was provided by the Australian Therapeutic Goods Administration.^18^ Bortezomib global approval was determined via personal communication with Takeda Oncology (email communication; Takeda Oncology; May 3, 2018). Data on stem cell transplant availability were reported using a prior publication by the World Bone Marrow Transplant Society.^19^ To estimate the effect of population growth on myeloma incidence, we applied the population size of 2016 onto the rate, sex, and age structure of 1990. To estimate the effect of aging on incident cases, we applied the age structure of 2016 onto the rate, sex distribution, and population size of 1990. To estimate the effect of changing age-specific incidence rates on the incident cases, we applied the incidence rates for 1990 onto the population size and age structure of 2016.

## Results

Data used for the MM estimates included 16 005 site-years from a vital registration system and 2737 site-years from cancer registry data. In 2016 there were 138 509 (95% UI, 121 000-155 480) incident cases of MM, with an age-standardized incidence rate (ASIR) of 2.1 per 100 000 persons (95% UI, 1.8-2.3). Multiple myeloma was responsible for 98 437 (95% UI, 87 383-109 815) deaths globally with an age-standardized death rate (ASDR) of 1.5 per 100 000 persons (95% UI, 1.3-1.7). Multiple myeloma was responsible for 2.1 million (95% UI, 1.9-2.3 million) DALYs at the global level in 2016, with an age-standardized rate of 30.5 (95% UI, 27.4-33.9) DALYs per 100 000 person-years (eTable 3 in the Supplement). From 1990 to 2016, MM incident cases increased by 126%, and deaths increased by 94% (eTable 4 in the Supplement). Among SDI quintiles, the largest increase (192%) was seen in middle SDI countries (from 7974 [95% UI, 7233-8821] in 1990 to 23 273 [95% UI, 21 136-26 947] in 2016). Of the 126% increase in incident cases at the global level, population growth contributed 40.4%, an aging world population contributed 52.9%, and increases in age-specific incidence rates contributed 32.6% (eTable 4 in the Supplement). Among the regions, the largest increase in incident cases from 1990 to 2016 was seen in East Asia (China, North Korea, and Taiwan), with a rise of 262% (from 4760 [95% UI, 4271-5575] in 1990 to 17 218 [95% UI, 14 482-19 093] in 2016). The largest contributor to this increase was a rise in age-specific incidence rates (contributing 157%), followed by an aging population (contributing 85%) and population growth (contributing 20%) (eTable 4 in the Supplement).

**Figure 1.**
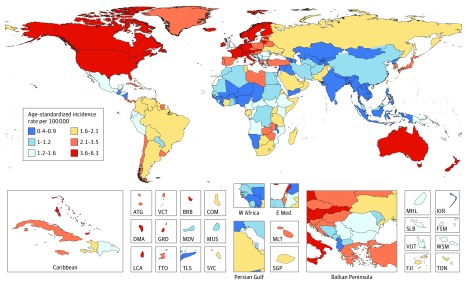
Age-Standardized Incidence Rate of Multiple Myeloma Age-standardized incidence rate of multiple myeloma, both sexes, 2016. ATG indicates Antigua and Barbuda; BRB, Barbados; COM, Comoros; DMA, Dominica; FJI, Fiji; FSM, Federated States of Micronesia; GRD, Grenada; KIR, Kiribati; LCA, Saint Lucia; MDV, Maldives; MHL, Marshall Islands; MLT, Malta; MUS, Mauritius; TLS, Timor-Leste; TON, Tonga; TTO, Trinidad and Tobago; SGP, Singapore; SLB, Solomon Islands; SYC, Seychelles; VCT, Saint Vincent and the Grenadines; VUT, Vanuatu; and WSM, Samo (Formerly Western Samoa).

The 3 world regions with the highest ASIR of MM were Australasia (5.8; 95% UI, 4.4-6.5), high-income North America (5.2; 95% UI, 4.7-6.5), and Western Europe (4.6; 95% UI, 3.7-5.5) (eTable 3 in the Supplement). The 3 highest ASDR were seen in high-income North America (3.0; 95% UI, 2.6-3.6), Australasia (2.8; 95% UI, 2.1-3.1), and Western Europe (2.6; 95% UI, 2.1-3.1). In terms of absolute numbers, Western Europe was the region with the most cases of myeloma for both sexes in 2016 (35 433; 95% UI, 28 272-42 151), followed by high-income North America (27 003; 95% UI, 24 157-33 637) and East Asia (17 218; 95% UI, 14 482-19 093). Most deaths occurred in Western Europe (22 060; 95% UI, 17 571-25 628), high-income North America (15 894; 95% UI, 14 059-19 364), and South Asia (11 187; 95% UI, 8975-12 182) (see eTable 3 in the Supplement). The countries with the most incident cases and deaths were the United States (24 407 [95% UI, 21 812-30 331] incident cases and 14 212 [95% UI, 12 523-17 316] deaths), China ([16 537; 95% UI, 14 094-18 617] incident cases and 10 363 [95% UI, 9079-11 898] deaths), and India (8 940 [95% UI, 7142-9710] incident cases and 8 715 [95% UI, 6990-9600] deaths) (incident cases and deaths: https://vizhub.healthdata.org/gbd-compare/). The ASIR and ASDR were the highest in high-income countries with an almost 10-fold difference between countries with the lowest and the highest ASIR and ASDR (Figure 1; eFigure 3 in the Supplement). From 1990 to 2016, ASIRs rose in all SDI quintiles except for the low SDI quintile, where they remained stable (eFigure 4 in the Supplement). However, ASDRs peaked in the high-SDI quintile around the year 2000 and have been declining since. The same trend can be seen in high-middle SDI countries, with a more recent peak in ASDRs around 2005 and declining rates since. ASDRs in the other SDI quintiles have been rising (eFigure 5 in the Supplement). All results presented herein can also be found online at https://vizhub.healthdata.org/gbd-compare/.

**Figure 2.**
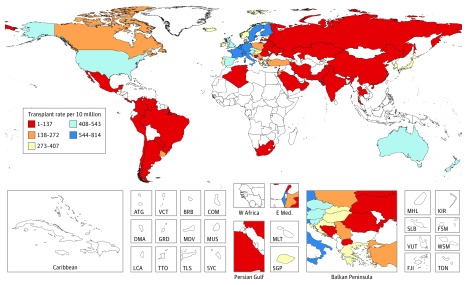
Stem-Cell Transplant Rate per 10 Million, 2010 Frequency of transplantation per 10 million people, both allogeneic and autologous transplant in 2010 as reported by Gratwohl et al.^19^ ATG indicates Antigua and Barbuda; BRB, Barbados; COM, Comoros; DMA, Dominica; FJI, Fiji; FSM, Federated States of Micronesia; GRD, Grenada; KIR, Kiribati; LCA, Saint Lucia; MDV, Maldives; MHL, Marshall Islands; MLT, Malta; MUS, Mauritius; TLS, Timor-Leste; TON, Tonga; TTO, Trinidad and Tobago; SGP, Singapore; SLB, Solomon Islands; SYC, Seychelles; VCT, Saint Vincent and the Grenadines; VUT, Vanuatu; and WSM, Samo (Formerly Western Samoa).

**Figure 3.**
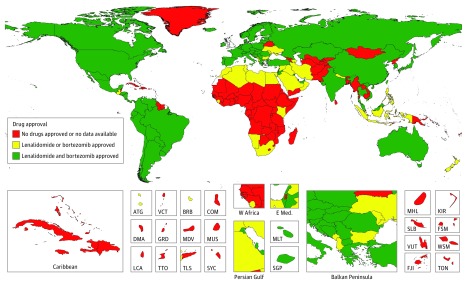
Lenalidomide and Bortezomib Approval, 2016 ATG indicates Antigua and Barbuda; BRB, Barbados; COM, Comoros; DMA, Dominica; FJI, Fiji; FSM, Federated States of Micronesia; GRD, Grenada; KIR, Kiribati; LCA, Saint Lucia; MDV, Maldives; MHL, Marshall Islands; MLT, Malta; MUS, Mauritius; TLS, Timor-Leste; TON, Tonga; TTO, Trinidad and Tobago; SGP, Singapore; SLB, Solomon Islands; SYC, Seychelles; VCT, Saint Vincent and the Grenadines; VUT, Vanuatu; and WSM, Samo (Formerly Western Samoa).

For stem cell transplant availability, the top 5 countries with the highest rates (per 10 million population) of stem cell transplantation (for all indications, not just MM) were Israel (814), Italy (671), Germany (665), Sweden (625), and the Netherlands (614) (Figure 2). Some regions of the world lack access to stem cell transplantation entirely, particularly sub-Saharan Africa (with the exception of South Africa). With respect to drug availability, as of 2016, out of 195 countries and territories, lenalidomide (Revlimid) had been approved in 73 countries and bortezomib (Velcade) in 103 countries (Figure 3). Notably, neither drug has been approved in most countries in sub-Saharan Africa and some countries in Central Asia.

## Discussion

Age-standardized incidence and death rates are highest in the Australasian, North American, and Western European populations. The world regions with the lowest age-standardized incidence of MM are Asia, Oceania, and sub-Saharan Africa. These results are in line with those of prior studies that have shown that the reported incidence for hematologic malignant neoplasms is higher in high-income countries compared with LMIC^20,21^ With respect to these differences between incidence of MM in high-income and LMIC, a full discussion of the predisposition for myeloma is beyond the scope of this report, but known risk factors include antecedent diagnosis of monoclonal gammopathy of undetermined significance or smoldering myeloma, and possible occupational risk factors.^22^ In addition, there have been recently described autosomal germline mutations that can predispose a patient to MM.^23^ At least some of the differences in incidence may be due to lack of diagnostic abilities in lower SDI countries compared with high SDI countries and do not necessarily reflect differences in disease biology (eFigure 6 in the Supplement shows a map of the SDI quintiles). Supporting this hypothesis is the fact that the disease burden of MM in African Americans in the United States is markedly higher than that of European Americans in the United States, yet the disease burden among Africans living in Africa is among the lowest in the world, as shown in our study and in previous studies.^24,25^ To explain the heterogeneity in incidence, collaborative research within the global oncology community is required on 2 fronts: the basic research question of differences in underlying disease biology, and the population health question of underascertainment. Despite the heterogeneous geographical burden of myeloma, time trends show that incident cases of myeloma have increased in all regions and have more than doubled for all SDI quintiles from 1990 to 2016. Global incident cases of myeloma increased by 126%, with aging contributing 52.9%. This is in line with the increase seen for other cancers that predominantly affect an older population. Prostate cancer cases at the global level, for example, increased by 217% from 1990 to 2013.^26^ We also showed that among SDI quintiles, the largest increase of 192% in incidence cases was seen in middle-SDI countries with an aging population and rising age-specific rates contributing equally. Among regions, the largest increase of 262% was seen in East Asia, mostly from a rise in age-specific incidence rates. Given the lack of known strong risk factors for myeloma, research in locations with rapidly rising incidence rates might help to identify predisposing factors and guide future preventative strategies.

We have also described an improvement in ASDRs for MM from 1990 to 2016 in high-income SDI regions despite increasing incidence rates, which is likely reflective of improvements in treatments. Novel agents, such as PIs and IMIDs, in addition to high-dose melphalan and ASCT, have changed the therapeutic landscape, with improvement in 5-year survival rates from 25.1% in 1975 to 1979 to 52.7% in 2009 in the United States.^7,27^

With respect to availability of effective therapies, we described the approval status of bortezomib and lenalidomide and the availability of ASCT as a surrogate measure, although our ability to assess true “availability”—as determined by factors such as cost, affordability, and willingness of insurers or governments to pay for cancer treatments—is limited. Prior research has demonstrated major differences in affordability of important cancer drugs around the world—bortezomib was specifically examined in this analysis.^28^ Nonetheless, 2 drugs that are now considered standard of care, lenalidomide and bortezomib, have not been approved in some African and Middle Eastern countries, and it is notable that there are no stem cell transplant centers in sub-Saharan Africa, with the exception of South Africa. Thus, on a global level there are marked discrepancies in the availability of effective therapies. In addition to ensuring universal access to health care, as specified in target 3.8 of the Sustainable Development Goals, it is imperative to at least ensure access to highly effective medications.^29^ Initiatives like the market access agreements negotiated by the American Cancer Society and the Clinton Health Access Initiative with Pfizer Inc and Cipla Inc to expand access to essential cancer medications are hopefully reflective of improved affordability, as has been the case with antiretroviral treatments for HIV/AIDS.^30^

### Limitations

Limitations of our study are that data informing the models to estimate the burden of myeloma are often scarce, especially in LMICs where vital registration systems and cancer registries are lacking or cover only a small part of the population. The uncertainty around our estimates in these locations is therefore large. Supporting the development of cancer registries and expanding vital registration systems in these areas are crucial. Unfortunately, our attempts to obtain data on drug sales from pharmaceutical companies were unsuccessful, and therefore we could report only drug availability. Given the challenges of estimating access to generic drugs like thalidomide, we chose to report approval rates of lenalidomide and bortezomib with the understanding that thalidomide might represent an effective treatment options in countries that have not approved lenalidomide. Other questions, such as which drugs are available through government-sponsored health plans, which phases of the disease the drug is approved in, which combinations are approved, were not specifically examined, and thus we were limited in making more definitive conclusions about drug “access” beyond regulatory approval. As an important example, within the European Union (EU), the European Medicines Agency (EMA) is responsible for the authorization of medicines in EU countries. The EMA has approved lenalidomide for use in the EU; however, member countries have individually decided whether the drug will be paid for by governmental health insurance. Thus, although marketing approval may be present, it does not guarantee that a drug will be covered by insurances or that patients can afford the cost. With respect to transplantation, we reported prior data analyzing global availability of transplantation but did not have access to myeloma-specific transplant rates. Further research is needed to fully describe details on the availability, access, and reimbursement of effective treatments for myeloma.

## Conclusions

Although more common in high SDI countries, myeloma is a global disease, and there has been a marked increase in incident cases from 1990 to 2016, predominantly in middle SDI countries and East Asia. Approval for effective drugs and stem cell transplantation options are lacking in many low-SDI countries. Collaborative global efforts are needed to ensure that every patient with myeloma is being diagnosed and has access to effective treatment. Further research is needed to determine the reasons behind the observed heterogeneity in disease burden.

## Notes

Supplement. eTable 1: Covariates selected for CODEm for multiple myeloma and expected direction of covariate eTable 2: Disability weights eTable 3: 2016 Multiple myeloma incidence and deaths at the global level, by region, and by SDI quintile eTable 4: Decomposition analysis of multiple myeloma incidence trends at the global and regional levels, and by SDI quintiles, both sexes, 1990 to 2016 eFigure 1: Flowchart describing estimation steps for multiple myeloma mortality and YLLs eFigure 2: Flowchart describing estimation steps for multiple myeloma incidence, prevalence, and YLDs eFigure 3: Age-standardized mortality rate of multiple myeloma, both sexes, 2016 eFigure 4: Trends in age-standardized incidence rate of multiple myeloma by Socio-demographic Index, both sexes, 1990–2016 eFigure 5: Trends in age-standardized mortality rate of multiple myeloma by Socio-demographic Index, both sexes, 1990–2016 eFigure 6: Sociodemographic Index quintiles, 2016

## References

[1] Kazandjian D, Landgren O A look backward and forward in the regulatory and treatment history of multiple myeloma: approval of novel-novel agents, new drug development, and longer patient survival. Semin Oncol. 2016;43(6):682-689. 28061986 10.1053/j.seminoncol.2016.10.008 PMC5282737

[2] Raju GK, Gurumurthi K, Domike R, A benefit-risk analysis approach to capture regulatory decision-making: multiple myeloma. Clin Pharmacol Ther. 2018;103(1):67-76. 28901535 10.1002/cpt.871 PMC7418461

[3] Fleming KA, Naidoo M, Wilson M, An essential pathology package for low- and middle-income countries. Am J Clin Pathol. 2017;147(1):15-32. 28158414 10.1093/ajcp/aqw143

[4] Tan D, Chng WJ, Chou T, Management of multiple myeloma in Asia: resource-stratified guidelines. Lancet Oncol. 2013;14(12):e571-e581.. 24176575 10.1016/S1470-2045(13)70404-2

[5] Attal M, Lauwers-Cances V, Hulin C, ; IFM 2009 Study Lenalidomide, bortezomib, and dexamethasone with transplantation for myeloma. N Engl J Med. 2017;376(14):1311-1320. 28379796 10.1056/NEJMoa1611750 PMC6201242

[6] McCarthy PL, Holstein SA, Petrucci MT, Lenalidomide maintenance after autologous stem-cell transplantation in newly diagnosed multiple myeloma: a meta-analysis. J Clin Oncol. 2017;35(29):3279-3289. 28742454 10.1200/JCO.2017.72.6679 PMC5652871

[7] Rajkumar SV Multiple myeloma: 2016 update on diagnosis, risk-stratification, and management. Am J Hematol. 2016;91(7):719-734. 27291302 10.1002/ajh.24402 PMC5291298

[8] Omoti CE, Omuemu CE Multiple myeloma: a ten-year study of survival and therapy in a developing nation. J Pak Med Assoc. 2007;57(7):341-344. 17867255

[9] Nwabuko OC, Igbigbi EE, Chukwuonye II, Nnoli MA Multiple myeloma in Niger Delta, Nigeria: complications and the outcome of palliative interventions. Cancer Manag Res. 2017;9:189-196. 28579833 10.2147/CMAR.S126136 PMC5446965

[10] GBD 2016 Causes of Death Collaborators Global, regional, and national age-sex specific mortality for 264 causes of death, 1980-2016: a systematic analysis for the Global Burden of Disease Study 2016. Lancet. 2017;390(10100):1151-1210. 28919116 10.1016/S0140-6736(17)32152-9 PMC5605883

[11] GBD 2016 DALYs and HALE Collaborators Global, regional, and national disability-adjusted life-years (DALYs) for 333 diseases and injuries and healthy life expectancy (HALE) for 195 countries and territories, 1990-2016: a systematic analysis for the Global Burden of Disease Study 2016. Lancet. 2017;390(10100):1260-1344. 28919118 10.1016/S0140-6736(17)32130-X PMC5605707

[12] Fonseca R, Abouzaid S, Bonafede M, Trends in overall survival and costs of multiple myeloma, 2000-2014. Leukemia. 2017;31(9):1915-1921. 28008176 10.1038/leu.2016.380 PMC5596206

[13] Disease GBD, Injury I, Prevalence C; GBD 2016 Disease and Injury Incidence and Prevalence Collaborators Global, regional, and national incidence, prevalence, and years lived with disability for 328 diseases and injuries for 195 countries, 1990-2016: a systematic analysis for the Global Burden of Disease Study 2016. Lancet. 2017;390(10100):1211-1259. 28919117 10.1016/S0140-6736(17)32154-2 PMC5605509

[14] Foreman KJ, Lozano R, Lopez AD, Murray CJ Modeling causes of death: an integrated approach using CODEm. Popul Health Metr. 2012;10:1. 22226226 10.1186/1478-7954-10-1 PMC3315398

[15] Neal RD, Din NU, Hamilton W, Comparison of cancer diagnostic intervals before and after implementation of NICE guidelines: analysis of data from the UK General Practice Research Database. Br J Cancer. 2014;110(3):584-592. 24366304 10.1038/bjc.2013.791 PMC3915139

[16] SEER*Stat Database: Incidence. SEER 18 Regs Research Data + Hurricane Katrina Impacted Louisiana Cases, November 2012 Sub (1973-2010 Varying). Linked To County Attributes. Total U.S., 1969-2011 Counties, National Cancer Institute, DCCPS, Surveillance Research Program, Surveillance Systems Branch. Released April 2013, based on the November 2012 submission [Internet]. https://www.seer.cancer.gov/. Accessed January 1, 2018.

[17] GBD 2013 Mortality and Causes of Death Collaborators Global, regional, and national age-sex specific all-cause and cause-specific mortality for 240 causes of death, 1990-2013: a systematic analysis for the Global Burden of Disease Study 2013. Lancet. 2015;385(9963):117-171. 25530442 10.1016/S0140-6736(14)61682-2 PMC4340604

[18] Australian Public Assessment Report for Lenalidomide, 2016, Therapeutic Goods Administration, used by permission of the Australian Government. https://www.tga.gov.au/sites/default/files/auspar-lenalidomide-160205.pdf. Accessed May 8, 2018.

[19] Gratwohl A, Pasquini MC, Aljurf M, ; Worldwide Network for Blood and Marrow Transplantation (WBMT) One million haemopoietic stem-cell transplants: a retrospective observational study. Lancet Haematol. 2015;2(3):e91-e100. 26687803 10.1016/S2352-3026(15)00028-9

[20] Ferlay J, Soerjomataram I, Dikshit R, Cancer incidence and mortality worldwide: sources, methods and major patterns in GLOBOCAN 2012. Int J Cancer. 2015;136(5):E359-E386. 25220842 10.1002/ijc.29210

[21] Miranda-Filho A, Piñeros M, Ferlay J, Soerjomataram I, Monnereau A, Bray F Epidemiological patterns of leukaemia in 184 countries: a population-based study. Lancet Haematol. 2018;5(1):e14-e24. 29304322 10.1016/S2352-3026(17)30232-6

[22] Perrotta C, Staines A, Codd M, Multiple Myeloma and lifetime occupation: results from the EPILYMPH study. J Occup Med Toxicol. 2012;7(1):25. 23241100 10.1186/1745-6673-7-25 PMC3557218

[23] Wei X, Calvo-Vidal MN, Chen S, Germline mutations in lysine specific demethylase 1 (LSD1/KDM1A) confer susceptibility to multiple myeloma. [published online March 20, 2018]. Cancer Res. 2018;canres.1900.2017. 29559475 10.1158/0008-5472.CAN-17-1900 PMC5955848

[24] Konstantinopoulos PA, Pantanowitz L, Dezube BJ Higher prevalence of monoclonal gammopathy of undetermined significance in African Americans than whites: the unknown role of underlying HIV infection. J Natl Med Assoc. 2006;98(11):1860-1861. 17128699 PMC2569800

[25] Landgren O, Gridley G, Turesson I, Risk of monoclonal gammopathy of undetermined significance (MGUS) and subsequent multiple myeloma among African American and white veterans in the United States. Blood. 2006;107(3):904-906. 16210333 10.1182/blood-2005-08-3449 PMC1895893

[26] Fitzmaurice C, Dicker D, Pain A, ; Global Burden of Disease Cancer Collaboration The global burden of cancer 2013. JAMA Oncol. 2015;1(4):505-527. 26181261 10.1001/jamaoncol.2015.0735 PMC4500822

[27] Howlader NNA, Krapcho M, Miller D, In: Cronin KA, ed. SEER Cancer Statistics Review, 1975-2014, National Cancer Institute. Bethesda, MD. https://seer.cancer.gov/archive/csr/1975_2014/. Based on November 2016 SEER data submission, posted to the SEER web site. Accessed January 1, 2018.

[28] Goldstein DA, Clark J, Tu Y, A global comparison of the cost of patented cancer drugs in relation to global differences in wealth. Oncotarget. 2017;8(42):71548-71555. 29069727 10.18632/oncotarget.17742 PMC5641070

[29] United Nations. Sustainable Development Goals. https://sustainabledevelopment.un.org/. Accessed April 7, 2018.

[30] McNeil DG Jr As cancer tears through Africa, drug makers draw up a battle plan. *The New York Times* https://www.nytimes.com/2017/10/07/health/africa-cancer-drugs.html. Accessed April 9, 2018.

