# Supplementary material for: Global Burden of Multiple Myeloma: A Systematic Analysis for the Global Burden of Disease Study 2016
Source: JAMA Oncol. 2018 May 16;4(9):1221–7. doi: 10.1001/jamaoncol.2018.2128 (PMC6143021; doi:10.1001/jamaoncol.2018.2128)
Supplement: Supplement. — eTable 1: Covariates selected for CODEm for multiple myeloma and expected direction of covariate eTable 2: Disability weights eTable 3: 2016 Multiple myeloma incidence and deaths at the global level, by region, and by SDI quintile eTable 4: Decomposition analysis of multiple myeloma incidence trends at the global and regional levels, and by SDI quintiles, both sexes, 1990 to 2016 eFigure 1: Flowchart describing estimation steps for multiple myeloma mortality and YLLs eFigure 2: Flowchart describing estimation steps for multiple myeloma incidence, prevalence, and YLDs eFigure 3: Age-standardized mortality rate of multiple myeloma, both sexes, 2016 eFigure 4: Trends in age-standardized incidence rate of multiple myeloma by Socio-demographic Index, both sexes, 1990–2016 eFigure 5: Trends in age-standardized mortality rate of multiple myeloma by Socio-demographic Index, both sexes, 1990–2016 eFigure 6: Sociodemographic Index quintiles, 2016 [file jamaoncol-4-1221-s001.pdf]

Supplementary Online Content

Cowan AJ, Allen C, Barac A, et al. Global burden of multiple myeloma: a systematic analysis for the Global Burden of Disease Study 2016. *JAMA Oncology*. Published online May 16, 2018. doi:10.1001/jamaoncol.2018.2128

eTable 1: Covariates selected for CODEm for multiple myeloma and expected direction of covariate

eTable 2: Disability weights

eTable 3: 2016 Multiple myeloma incidence and deaths at the global level, by region, and by SDI quintile

eTable 4: Decomposition analysis of multiple myeloma incidence trends at the global and regional levels, and by SDI quintiles, both sexes, 1990 to 2016

eFigure 1: Flowchart describing estimation steps for multiple myeloma mortality and YLLs

eFigure 2: Flowchart describing estimation steps for multiple myeloma incidence, prevalence, and YLDs

eFigure 3: Age-standardized mortality rate of multiple myeloma, both sexes, 2016

eFigure 4: Trends in age-standardized incidence rate of multiple myeloma by Socio-demographic Index, both sexes, 1990–2016

eFigure 5: Trends in age-standardized mortality rate of multiple myeloma by Socio-demographic Index, both sexes, 1990–2016

eFigure 6: Sociodemographic Index quintiles, 2016

This supplementary material has been provided by the authors to give readers additional information about their work.

© 2018 American Medical Association. All rights reserved.

Tables

eTable 1: Covariates selected for CODEm for multiple myeloma and expected direction of covariate

Cause	***Sex***	***Age start***	***Age end***	***Direction***	***Covariate***
Multiple myeloma	*Both*	*15-19 years*	*95+ years*	*1*	*Alcohol (liters per capita)*
Multiple myeloma	*Both*	*15-19 years*	*95+ years*	*1*	*Tobacco (cigarettes per capita)*
Multiple myeloma	*Both*	*15-19 years*	*95+ years*	*-1*	*Education (years per capita)*
Multiple myeloma	*Both*	*15-19 years*	*95+ years*	*0*	*LDI (I$ per capita)*
Multiple myeloma	*Both*	*15-19 years*	*95+ years*	*1*	*Mean BMI*
Multiple myeloma	*Both*	*15-19 years*	*95+ years*	*-1*	*Sanitation (proportion with access)*
Multiple myeloma	*Both*	*15-19 years*	*95+ years*	*1*	*Smoking Prevalence*
Multiple myeloma	*Both*	*15-19 years*	*95+ years*	*-1*	*Improved Water Source (proportion with access)*
Multiple myeloma	*Both*	*15-19 years*	*95+ years*	*0*	*Socio-demographic Index*
Multiple myeloma	*Both*	*15-19 years*	*95+ years*	*-1*	*fruits adjusted(g)*
Multiple myeloma	*Both*	*15-19 years*	*95+ years*	*1*	*red meats adjusted(g)*
Multiple myeloma	*Both*	*15-19 years*	*95+ years*	*-1*	*vegetables adjusted(g)*
Multiple myeloma	*Both*	*15-19 years*	*95+ years*	*-1*	*Healthcare access and quality index*

© 2018 American Medical Association. All rights reserved.

eTable 2: Disability weights

Health state	***Lay description***	***Estimate***	***Uncertainty interval***	
Cancer, diagnosis and primary therapy	*Has pain, nausea, fatigue, weight loss and high anxiety.*	*0.288*	*0.193*	*0.399*
Cancer, controlled phase	*Has a chronic disease that requires medication every day and causes some worry but minimal interference with daily activities.*	*0.049*	*0.031*	*0.072*
Cancer, metastatic	*Has severe pain, extreme fatigue, weight loss and high anxiety.*	*0.451*	*0.307*	*0.600*
Terminal phase, with medication	*Has lost a lot of weight and regularly uses strong medication to avoid constant pain. The person has no appetite, feels nauseous, and needs to spend most of the day in bed.*	*0.540*	*0.377*	*0.687*

© 2018 American Medical Association. All rights reserved.

eTable 3: 2016 Multiple myeloma incidence and deaths at the global level, by region, and by SDI quintile

Location	Incidence cases, global			ASIR, both sexes (per 100 000)			Deaths, global			ASDR, both sexes (per 100 000)		
	Male	Female	Both	Male	Female	Both	Male	Female	Both	Male	Female	Both
Global	74539 (59713 to 88383)	63971 (51690 to 77467)	138509 (119064 to 158268)	2.4 (1.9 to 2.9)	1.8 (1.4 to 2.2)	2.1 (1.8 to 2.4)	51037 (40967 to 60242)	47400 (38565 to 56364)	98437 (85269 to 111286)	1.7 (1.4 to 2.0)	1.3 (1.1 to 1.6)	1.5 (1.3 to 1.7)
High SDI	40133 (29610 to 50738)	33588 (25234 to 43889)	73721 (59796 to 89358)	5.2 (3.9 to 6.6)	3.5 (2.6 to 4.6)	4.3 (3.5 to 5.2)	23912 (17440 to 29624)	21010 (15645 to 27051)	44922 (36135 to 53655)	3.0 (2.2 to 3.8)	2.0 (1.5 to 2.6)	2.4 (2.0 to 3.0)
High-middle SDI	13593 (10662 to 16055)	11708 (9186 to 14465)	25301 (21475 to 28785)	2.3 (1.8 to 2.8)	1.7 (1.3 to 2.0)	2.0 (1.7 to 2.2)	8475 (6774 to 10167)	7966 (6378 to 10001)	16441 (14211 to 19048)	1.5 (1.2 to 1.8)	1.1 (0.9 to 1.4)	1.3 (1.1 to 1.5)
Low SDI	1740 (1178 to 2128)	2312 (1668 to 2667)	4052 (3055 to 4646)	1.1 (0.7 to 1.4)	1.3 (1.0 to 1.5)	1.2 (0.9 to 1.4)	2094 (1442 to 2561)	2748 (1982 to 3262)	4842 (3703 to 5574)	1.4 (0.9 to 1.7)	1.7 (1.2 to 2.0)	1.5 (1.2 to 1.8)
Low-middle SDI	6192 (4719 to 7597)	6333.0 (5042.0 to 7610.0)	12525 (10515 to 14383)	0.9 (0.7 to 1.1)	0.9 (0.7 to 1.1)	0.9 (0.8 to 1.1)	5751 (4434 to 7200)	6419 (5085 to 7851)	12170 (10180 to 14214)	0.9 (0.7 to 1.1)	0.9 (0.7 to 1.2)	0.9 (0.8 to 1.1)
Middle SDI	13063 (10411 to 16759)	10210 (8613 to 12157)	23273 (20449 to 27400)	1.2 (1.0 to 1.6)	0.9 (0.8 to 1.1)	1.1 (0.9 to 1.2)	10773 (8584 to 13766)	9225 (7814 to 10996)	19998 (17541 to 23447)	1.1 (0.8 to 1.4)	0.8 (0.7 to 1.0)	0.9 (0.8 to 1.1)
High-income Asia Pacific	4314 (3281 to 5241)	4569 (3380 to 5852)	8882 (7153 to 10545)	2.6 (2.0 to 3.2)	2.1 (1.6 to 2.8)	2.4 (1.9 to 2.8)	2872 (2197 to 3530)	2857 (2085 to 3679)	5729 (4612 to 6844)	1.7 (1.3 to 2.1)	1.2 (0.9 to 1.6)	1.4 (1.2 to 1.7)
Western Europe	19385 (13366 to 24870)	16048 (11515 to 21222)	35433 (27371 to 43318)	5.7 (3.9 to 7.3)	3.7 (2.7 to 5.0)	4.6 (3.6 to 5.7)	11684 (8024 to 14724)	10376 (7494 to 13728)	22060 (17133 to 26667)	3.3 (2.3 to 4.1)	2.2 (1.6 to 2.9)	2.6 (2.1 to 3.2)
Andean Latin America	323 (258 to 423)	376 (299 to 513)	699 (594 to 884)	1.6 (1.2 to 2.0)	1.6 (1.3 to 2.2)	1.6 (1.3 to 2.0)	317 (239 to 427)	368 (278 to 515)	685 (558 to 875)	1.6 (1.2 to 2.1)	1.6 (1.2 to 2.2)	1.6 (1.3 to 2.0)
Central Latin America	1477 (1109 to 1955)	1662 (1365 to 2333)	3140 (2699 to 4015)	1.6 (1.2 to 2.1)	1.6 (1.3 to 2.2)	1.6 (1.4 to 2.0)	1287 (961 to 1690)	1445 (1184 to 2007)	2732 (2319 to 3474)	1.4 (1.1 to 1.9)	1.4 (1.2 to 1.9)	1.4 (1.2 to 1.8)
Southern Latin America	832 (607 to 1064)	796 (590 to 1032)	1629 (1333 to 1970)	2.7 (1.9 to 3.4)	1.9 (1.4 to 2.5)	2.3 (1.8 to 2.7)	724 (518 to 954)	715 (524 to 944)	1439 (1158 to 1753)	2.4 (1.7 to 3.1)	1.7 (1.2 to 2.2)	2.0 (1.6 to 2.4)

© 2018 American Medical Association. All rights reserved.

Location	Incidence cases, global			ASIR, both sexes (per 100 000)			Deaths, global			ASDR, both sexes (per 100 000)		
	Male	Female	Both	Male	Female	Both	Male	Female	Both	Male	Female	Both
Tropical Latin America	1844 (1323 to 2347)	1696 (1182 to 2041)	3540 (2722 to 4133)	2.1 (1.5 to 2.7)	1.6 (1.1 to 1.9)	1.8 (1.4 to 2.1)	1650 (1174 to 2076)	1515 (1058 to 1832)	3165 (2428 to 3700)	2.0 (1.4 to 2.5)	1.4 (1.0 to 1.7)	1.7 (1.3 to 1.9)
North Africa and Middle East	2954 (2303 to 4477)	2283 (1818 to 3196)	5238 (4415 to 7201)	1.7 (1.3 to 2.7)	1.2 (1.0 to 1.8)	1.5 (1.2 to 2.1)	2634 (2038 to 3939)	1966 (1545 to 2773)	4600 (3818 to 6283)	1.6 (1.3 to 2.5)	1.1 (0.9 to 1.6)	1.3 (1.1 to 1.9)
High-income North America	15281 (12011 to 20531)	11722 (9293 to 16225)	27003 (23224 to 34540)	6.5 (5.1 to 8.7)	4.1 (3.3 to 5.7)	5.2 (4.5 to 6.6)	8711 (6789 to 11470)	7183 (5651 to 9639)	15894 (13547 to 19876)	3.7 (2.9 to 4.9)	2.4 (1.9 to 3.2)	3.0 (2.5 to 3.7)
Oceania	32 (22 to 51)	50 (33 to 69)	82 (60 to 105)	1.3 (0.9 to 1.9)	1.5 (1.0 to 2.1)	1.4 (1.0 to 1.8)	28 (18 to 44)	45 (29 to 64)	72 (52 to 96)	1.1 (0.7 to 1.7)	1.4 (0.9 to 2.0)	1.3 (0.9 to 1.6)
Central sub-Saharan Africa	226 (160 to 318)	295 (212 to 380)	522 (402 to 651)	1.1 (0.8 to 1.5)	1.3 (0.9 to 1.6)	1.2 (0.9 to 1.5)	251 (176 to 349)	349 (246 to 463)	600 (456 to 754)	1.3 (0.9 to 1.8)	1.6 (1.1 to 2.1)	1.4 (1.1 to 1.8)
Eastern sub-Saharan Africa	1062 (650 to 1299)	1475 (939 to 1817)	2537 (1722 to 2991)	1.4 (0.9 to 1.7)	1.7 (1.1 to 2.1)	1.6 (1.1 to 1.8)	1264 (770 to 1587)	1699 (1129 to 2152)	2962 (2085 to 3539)	1.7 (1.1 to 2.1)	2.1 (1.4 to 2.6)	1.9 (1.3 to 2.3)
Central Asia	236 (189 to 307)	227 (179 to 293)	463 (394 to 567)	0.8 (0.6 to 1.0)	0.6 (0.5 to 0.8)	0.7 (0.6 to 0.8)	196 (155 to 256)	190 (149 to 250)	386 (326 to 477)	0.7 (0.5 to 0.9)	0.5 (0.4 to 0.7)	0.6 (0.5 to 0.7)
Southern sub-Saharan Africa	456 (322 to 555)	454 (308 to 550)	910 (688 to 1049)	2.3 (1.7 to 2.8)	1.7 (1.2 to 2.1)	2.0 (1.5 to 2.3)	430 (312 to 544)	442 (297 to 552)	872 (669 to 1023)	2.3 (1.7 to 3.0)	1.7 (1.2 to 2.1)	2.0 (1.5 to 2.3)
Western sub-Saharan Africa	599 (437 to 796)	955 (660 to 1257)	1553 (1188 to 1945)	0.8 (0.6 to 1.1)	1.1 (0.8 to 1.4)	1.0 (0.7 to 1.2)	728 (521 to 1019)	1131 (779 to 1514)	1860 (1424 to 2384)	1.1 (0.8 to 1.5)	1.4 (1.0 to 1.9)	1.3 (1.0 to 1.6)
East Asia	10901 (7786 to 13459)	6317 (4831 to 7779)	17218 (13909 to 19959)	1.3 (1.0 to 1.7)	0.8 (0.6 to 0.9)	1.0 (0.8 to 1.2)	6348 (4724 to 7998)	4481 (3508 to 5514)	10830 (9043 to 12580)	0.8 (0.6 to 1.0)	0.5 (0.4 to 0.7)	0.7 (0.6 to 0.8)
South Asia	6029 (3936 to 7182)	5696 (4287 to 6790)	11725 (8909 to 13337)	1.0 (0.7 to 1.2)	1.0 (0.7 to 1.2)	1.0 (0.8 to 1.1)	5558 (3659 to 6666)	5629 (4210 to 6839)	11187 (8567 to 12844)	1.0 (0.7 to 1.2)	1.0 (0.7 to 1.2)	1.0 (0.8 to 1.1)
Southeast Asia	2153 (1739 to 2977)	1964 (1639 to 2442)	4118 (3579 to 5213)	0.9 (0.7 to 1.2)	0.7 (0.6 to 0.9)	0.8 (0.7 to 1.0)	1986 (1601 to 2794)	2005 (1659 to 2521)	3991 (3448 to 5128)	0.8 (0.7 to 1.2)	0.8 (0.6 to 0.9)	0.8 (0.7 to 1.0)
Australasia	1368 (866	1009	2377 (1743	7.1 (4.5	4.6 (3.2	5.8 (4.2	687 (439	520 (363	1207	3.5 (2.2	2.2 (1.5	2.8 (2.1 to

© 2018 American Medical Association. All rights reserved.

Location	*Incidence cases, global*			*ASIR, both sexes (per 100 000)*			*Deaths, global*			*ASDR, both sexes (per 100 000)*		
	Male	***Female***	***Both***	***Male***	***Female***	***Both***	***Male***	***Female***	***Both***	***Male***	***Female***	***Both***
	to 1777)	*(706 to 1333)*	*to 2903)*	*to 9.3)*	*to 6.1)*	*to 7.1)*	*to 898)*	*to 682)*	*(888 to 1471)*	*to 4.6)*	*to 2.9)*	*3.4)*
Caribbean	*600 (473 to 722)*	*578 (440 to 681)*	*1178 (983 to 1327)*	*2.9 (2.3 to 3.5)*	*2.5 (1.9 to 2.9)*	*2.7 (2.2 to 3.0)*	*503 (395 to 616)*	*488 (369 to 585)*	*991 (821 to 1129)*	*2.5 (1.9 to 3.0)*	*2.1 (1.6 to 2.5)*	*2.2 (1.9 to 2.6)*
Central Europe	*1962 (1360 to 2401)*	*2074 (1416 to 2516)*	*4036 (3026 to 4688)*	*2.5 (1.7 to 3.0)*	*2.0 (1.4 to 2.4)*	*2.2 (1.7 to 2.6)*	*1596 (1103 to 1942)*	*1630 (1114 to 1961)*	*3226 (2415 to 3725)*	*2.0 (1.4 to 2.5)*	*1.5 (1.0 to 1.8)*	*1.7 (1.3 to 2.0)*
Eastern Europe	*2502 (1817 to 3384)*	*3727 (2504 to 5159)*	*6228 (4778 to 7971)*	*2.1 (1.5 to 2.9)*	*2.0 (1.3 to 2.8)*	*2.0 (1.6 to 2.6)*	*1551 (1033 to 2280)*	*2334 (1469 to 3495)*	*3885 (2846 to 5295)*	*1.3 (0.9 to 2.0)*	*1.2 (0.8 to 1.8)*	*1.3 (0.9 to 1.7)*

© 2018 American Medical Association. All rights reserved.

eTable 4: Decomposition analysis of multiple myeloma incidence trends at the global and regional levels, and by SDI quintiles, both sexes, 1990 to 2016

Location	***Incident cases, No.***		***Expected incident cases, 2016, No.***		***Change in incident cases, 1990 to 2016, %***			***Overall change, %***
	1990	***2016***	***Given population growth alone***	***Given population growth and aging***	***Due to population growth[a]***	***Due to change in age structure[b]***	***Due to change in incidence rate[c]***	
Global	*61307 (54273 to 72475)*	*138509 (119063 to 158267)*	*86083*	*118543*	*40.4*	*52.9*	*32.6*	*125.9*
High SDI	*35798 (30502 to 44220)*	*73720 (59795 to 89357)*	*41421*	*58781*	*15.7*	*48.5*	*41.7*	*105.9*
High-middle SDI	*10580 (9142 to 13275)*	*25300 (21475 to 28785)*	*13963*	*18710*	*32*	*44.9*	*62.3*	*139.1*
Middle SDI	*7974 (7011 to 9142)*	*23273 (20449 to 27399)*	*10292*	*16801*	*29.1*	*81.6*	*81.2*	*191.8*
Low-middle SDI	*5158 (4017 to 6072)*	*12525 (10515 to 14382)*	*8176*	*10718*	*58.5*	*49.3*	*35*	*142.8*
Low SDI	*1937 (1334 to 2313)*	*4052 (3054 to 4646)*	*4051*	*4083*	*109.2*	*1.6*	*-1.6*	*109.2*
High-income Asia Pacific	*3540 (3087 to 4417)*	*8882 (7153 to 10544)*	*3788*	*7646*	*7*	*108.9*	*34.9*	*150.9*
Western Europe	*18131 (15920 to 23187)*	*35432 (27370 to 43317)*	*20364*	*26839*	*12.3*	*35.7*	*47.4*	*95.4*
Andean Latin America	*333 (267 to 399)*	*698 (594 to 884)*	*513*	*786*	*53.8*	*82*	*-26.4*	*109.4*
Central Latin America	*1048 (894 to 1262)*	*3139 (2699 to 4014)*	*1575*	*2526*	*50.3*	*90.7*	*58.5*	*199.5*
Southern Latin America	*758 (643 to 960)*	*1628 (1332 to 1969)*	*1021*	*1289*	*34.8*	*35.4*	*44.7*	*114.8*
Tropical Latin America	*995 (865 to 1337)*	*3540 (2721 to 4132)*	*1391*	*2496*	*39.8*	*110.9*	*104.8*	*255.5*
North Africa and Middle East	*1794 (1363 to 2646)*	*5237 (4414 to 7201)*	*3092*	*4113*	*72.4*	*56.9*	*62.6*	*192*
High-income North America	*13237 (10727 to 15768)*	*27002 (23224 to 34539)*	*17115*	*21871*	*29.3*	*35.9*	*38.8*	*104*

© 2018 American Medical Association. All rights reserved.

Location	Incident cases, No.		Expected incident cases, 2016, No.		Change in incident cases, 1990 to 2016, %			Overall change, %
	1990	2016	Given population growth alone	Given population growth and aging	Due to population growth[a]	Due to change in age structure[b]	Due to change in incidence rate[c]	
**Oceania**	27 (20 to 37)	82 (59 to 104)	47	63	69.2	59.4	67.1	195.7
**Central sub-Saharan Africa**	270 (194 to 339)	521 (401 to 651)	612	575	126.2	-13.7	-19.9	92.7
**Eastern sub-Saharan Africa**	1163 (736 to 1398)	2536 (1722 to 2990)	2437	2477	109.5	3.4	5.1	118
**Central Asia**	304 (232 to 359)	462 (394 to 567)	391	471	28.3	26.3	-2.8	51.8
**Southern sub-Saharan Africa**	422 (284 to 515)	909 (688 to 1048)	613	760	45.4	34.9	35.3	115.6
**Western sub-Saharan Africa**	807 (583 to 994)	1553 (1188 to 1944)	1645	1563	103.8	-10.2	-1.3	92.3
**East Asia**	4759 (4122 to 5829)	17217 (13908 to 19958)	5728	9761	20.4	84.7	156.6	261.7
**South Asia**	4549 (3324 to 5299)	11724 (8908 to 13336)	7058	9672	55.2	57.4	45.1	157.7
**Southeast Asia**	1524 (1311 to 1834)	4117 (3579 to 5212)	2182	3261	43.2	70.8	56.2	170.1
**Australasia**	906 (742 to 1112)	2377 (1743 to 2902)	1272	1726	40.5	50.1	71.8	162.3
**Caribbean**	536 (449 to 609)	1177 (983 to 1327)	687	986	28.2	55.7	35.7	119.6
**Central Europe**	2173 (1921 to 2765)	4035 (3026 to 4687)	2043	2966	-6	42.5	49.2	85.7
**Eastern Europe**	4023 (3049 to 5448)	6228 (4778 to 7971)	3840	4809	-4.5	24.1	35.3	54.8

[a]To estimate the effect of population growth we applied the population size of 2016 onto the rate, sex, and age structure of 1990.

[b] To estimate the effect of aging on incident cases we applied the age structure of 2016 onto the rate, sex distribution, and population size of 1990. The change in incident cases reported herein shows the proportion of the change in incident cases between 1990 and 2016 that can be attributed to the changing age structure of the population.

[c] To estimate the effect of changing incidence rates on the incident cases we applied the incidence rates for 1990 onto the population size and age structure of 2016. The change in incident cases reported herein shows the proportion of the change in incident cases between 1990 and 2016 that can be attributed to a change in incidence rates.

© 2018 American Medical Association. All rights reserved.

© 2018 American Medical Association. All rights reserved.

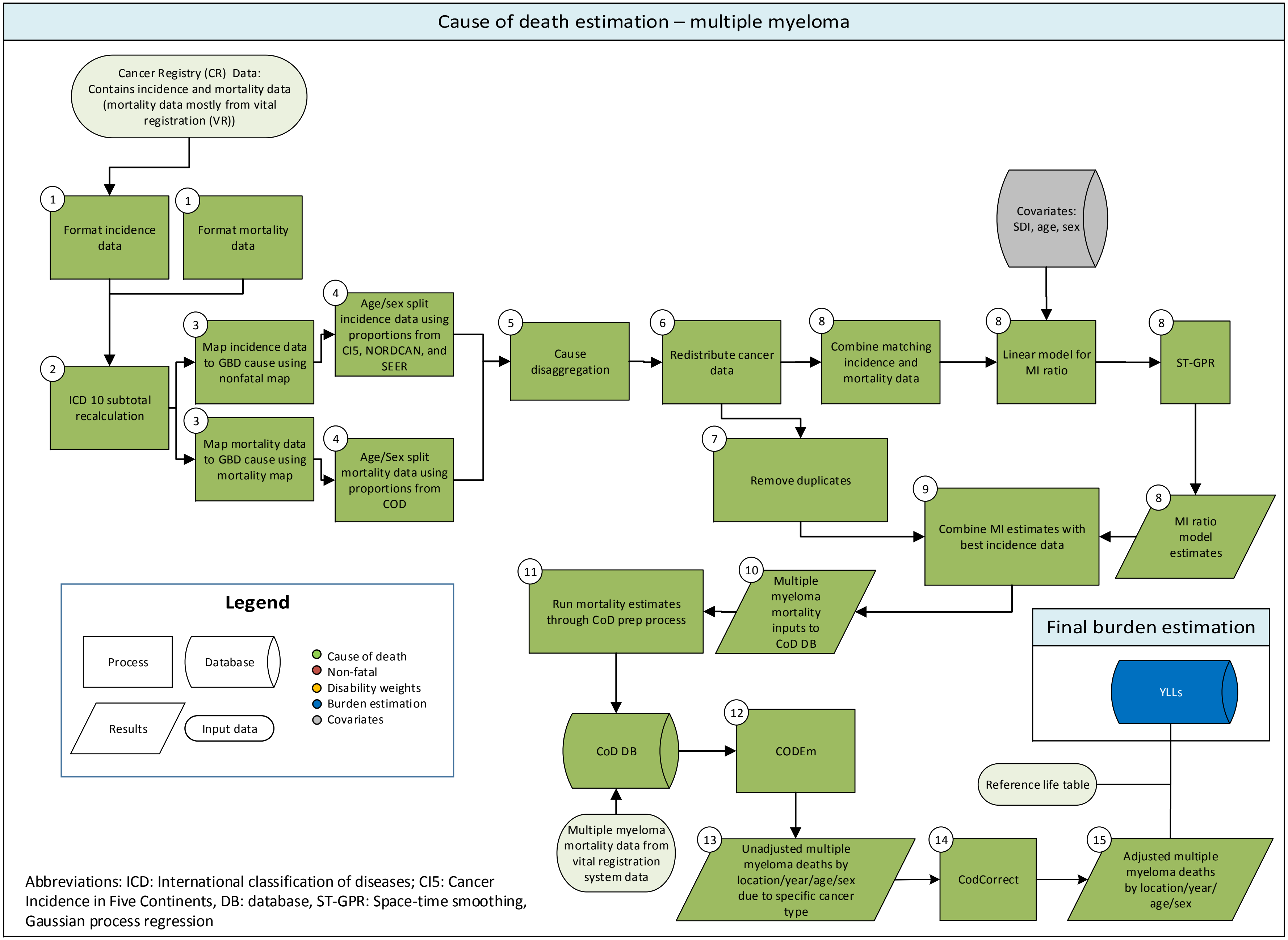

eFigure 1: Flowchart describing estimation steps for multiple myeloma mortality and YLLs

© 2018 American Medical Association. All rights reserved.

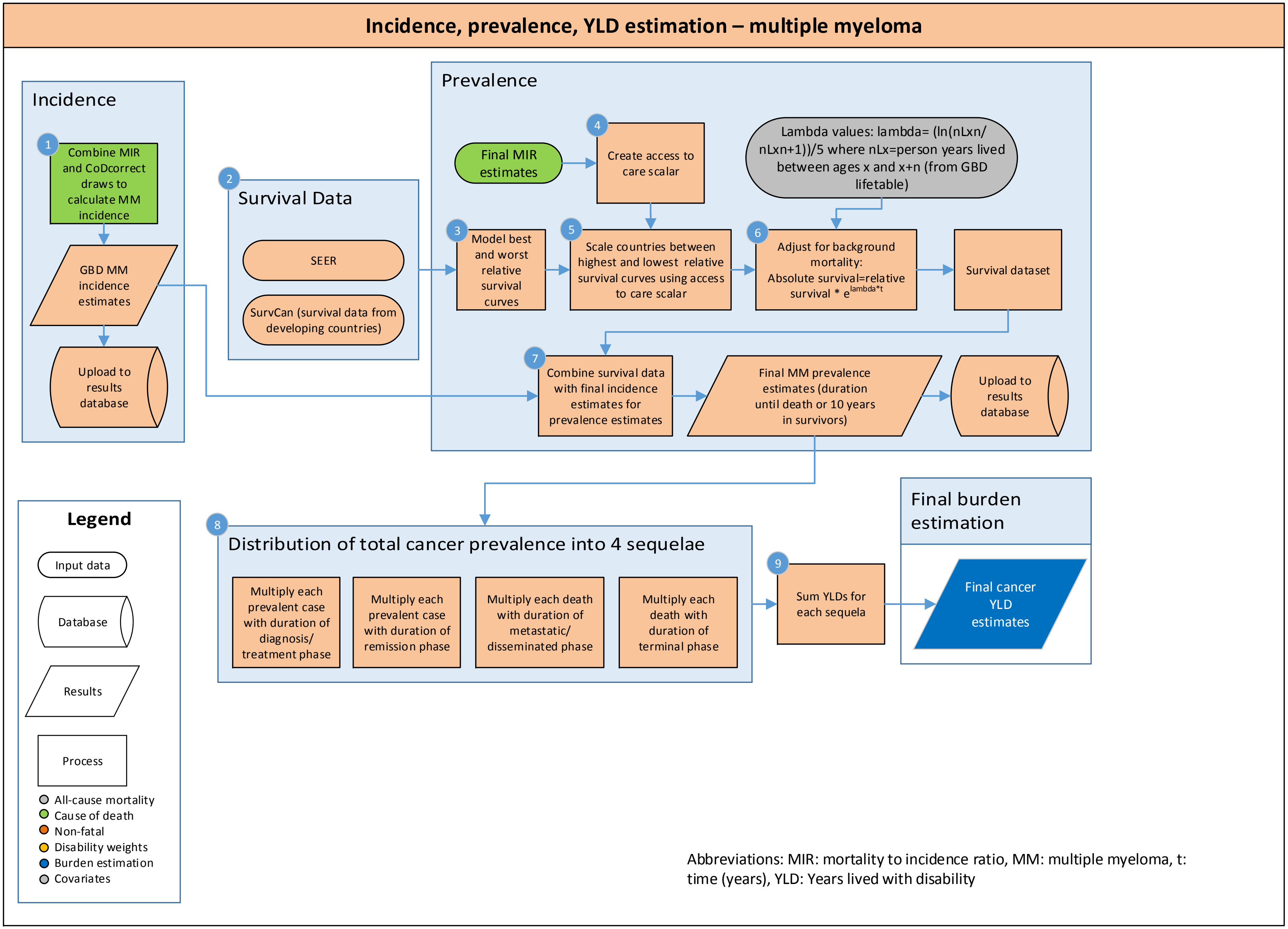

eFigure 2: Flowchart describing estimation steps for multiple myeloma incidence, prevalence, and YLDs

© 2018 American Medical Association. All rights reserved.

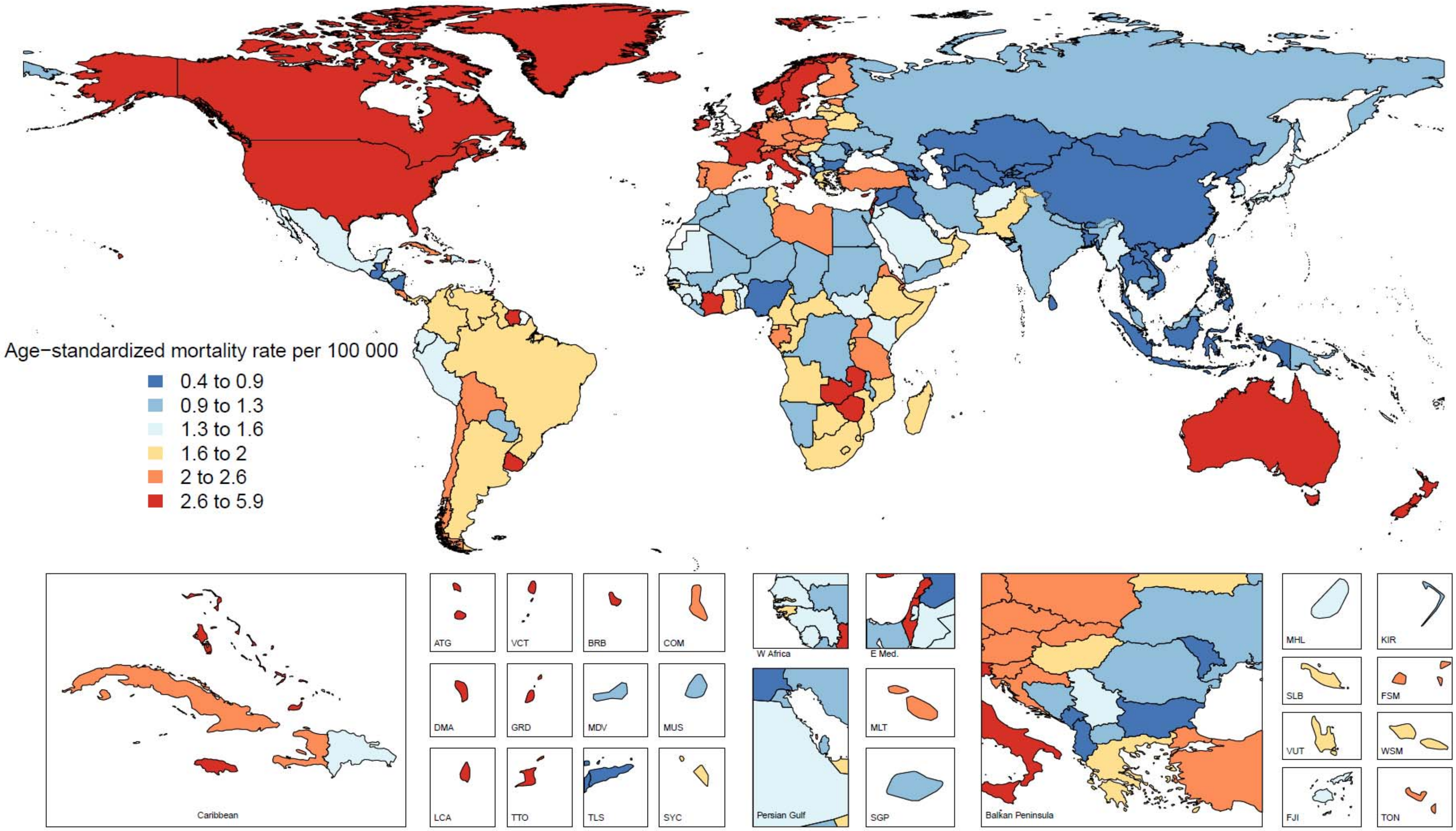

eFigure 3: Age-standardized mortality rate of multiple myeloma, both sexes, 2016

© 2018 American Medical Association. All rights reserved.

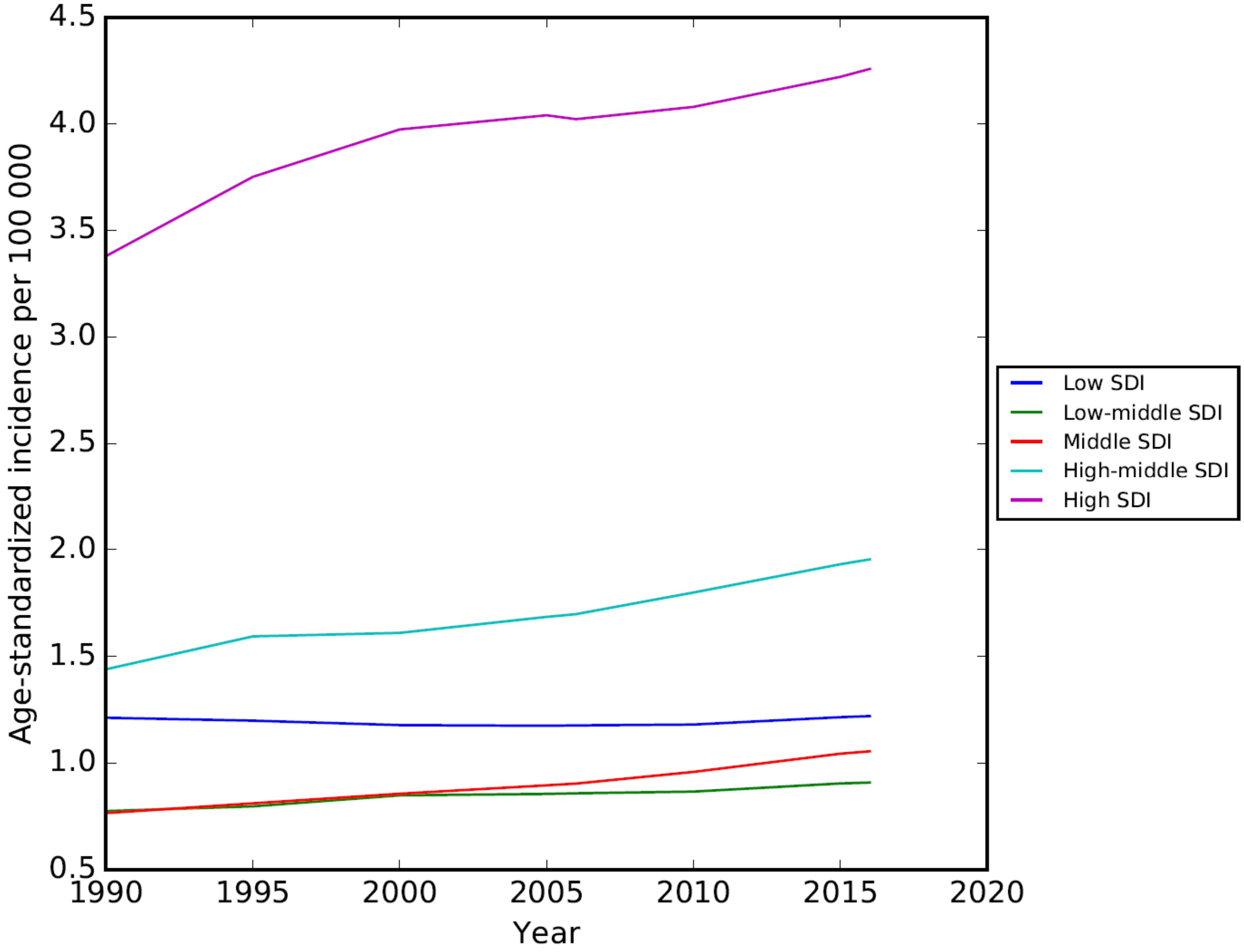

eFigure 4: Trends in age-standardized incidence rate of multiple myeloma by Socio-demographic Index, both sexes, 1990–2016

© 2018 American Medical Association. All rights reserved.

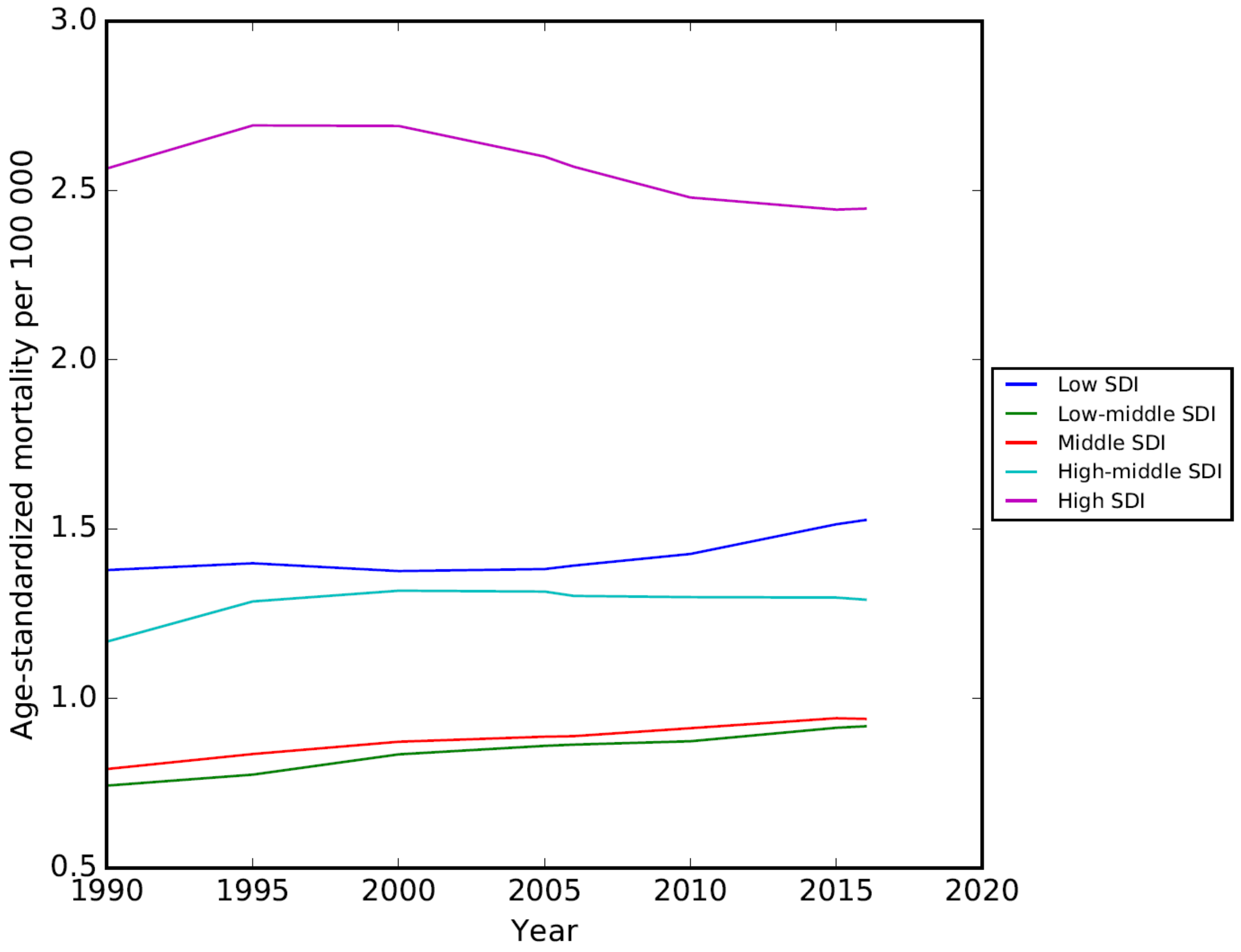

eFigure 5: Trends in age-standardized mortality rate of multiple myeloma by Socio-demographic Index, both sexes, 1990–2016

© 2018 American Medical Association. All rights reserved.

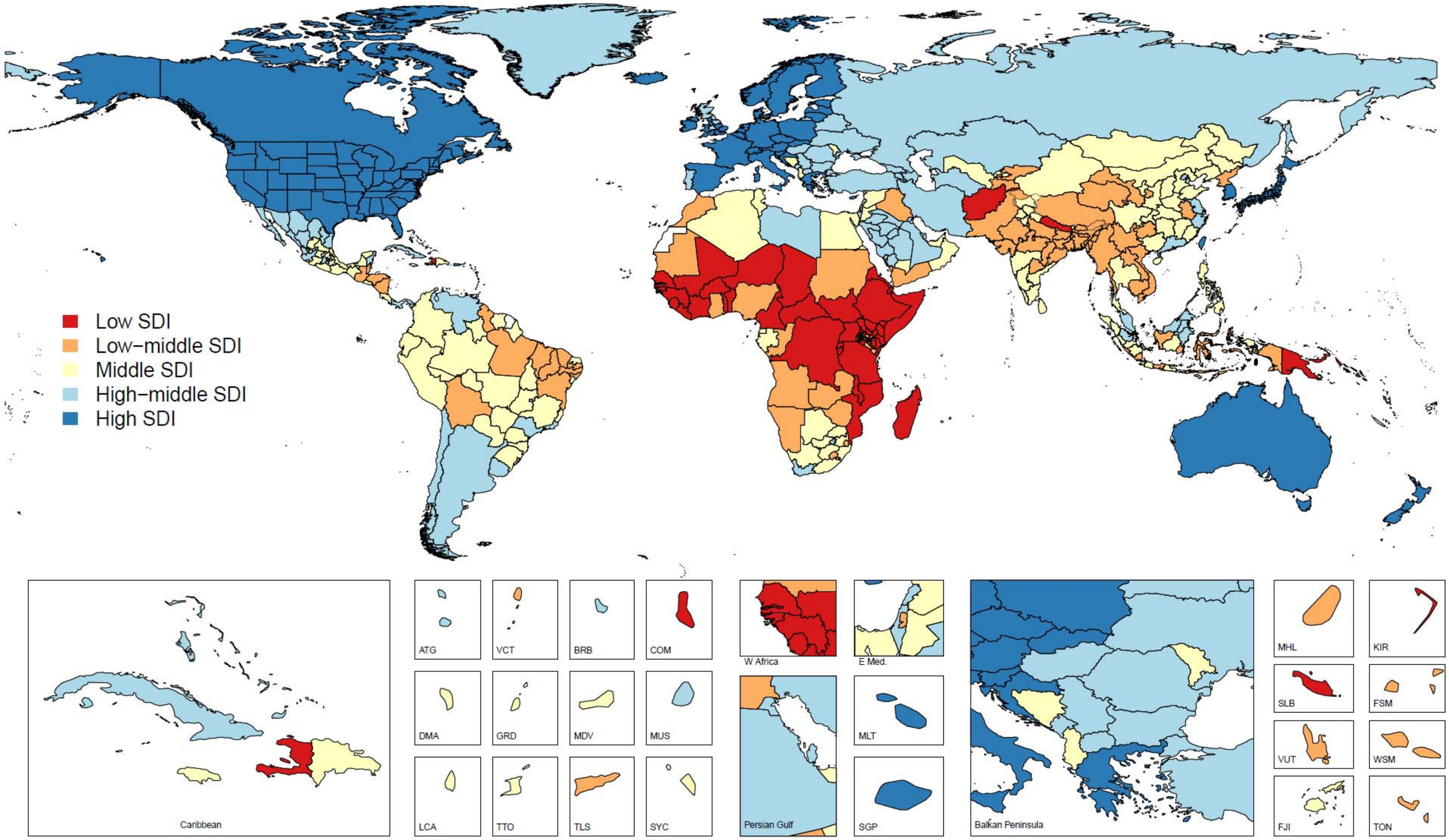

eFigure 6: Sociodemographic Index quintiles, 2016

© 2018 American Medical Association. All rights reserved.
